# Apolipoprotein(a) Inhibits *In Vitro* Tube Formation in Endothelial Cells: Identification of Roles for Kringle V and the Plasminogen Activation System

**DOI:** 10.1371/journal.pone.0052287

**Published:** 2013-01-11

**Authors:** Lei Liu, Michael B. Boffa, Marlys L. Koschinsky

**Affiliations:** 1 Department of Biochemistry, Queen's University, Kingston, Ontario, Canada; 2 Department of Chemistry and Biochemistry, University of Windsor, Windsor, Ontario, Canada; University of Frankfurt - University Hospital Frankfurt, Germany

## Abstract

Elevated plasma concentrations of lipoprotein(a) are associated with increased risk for atherothrombotic diseases. Apolipoprotein(a), the unique glycoprotein component of lipoprotein(a), is characterized by the presence of multiple kringle domains, and shares a high degree of sequence homology with the serine protease zymogen plasminogen. It has been shown that angiostatin, a proteolytic fragment of plasminogen containing kringles 1–4, can effectively inhibit angiogenesis. Moreover, proteolytic fragments of plasminogen containing kringle 5 are even more potent inhibitors of angiogenesis than angiostatin. Despite its strong similarity with plasminogen, the role of apolipoprotein(a) in angiogenesis remains controversial, with both pro- and anti-angiogenic effects reported. In the current study, we evaluated the ability of apolipoprotein(a) to inhibit VEGF- and angiopoietin-induced tube formation in human umbilical cord endothelial cells. A 17 kringle-containing form of recombinant apo(a) (17K), corresponding to a well-characterized, physiologically-relevant form of the molecule, effectively inhibited tube formation induced by either VEGF or angiopoietin-1. Using additional recombinant apolipoprotein(a) (r-apo(a)) variants, we demonstrated that this effect was dependent on the presence of an intact lysine-binding site in kringle V domain of apo(a), but not on the presence of the functional lysine-binding site in apo(a) kringle IV type 10; sequences within in the amino-terminal half of the molecule were also not required for the inhibitory effects of apo(a). We also showed that the apo(a)-mediated inhibition tube formation could be reversed, in part by the addition of plasmin or urokinase plasminogen activator, or by removal of plasminogen from the system. Further, we demonstrated that apo(a) treated with glycosidases to remove sialic acid was significantly less effective in inhibiting tube formation. This is the first report of a functional role for the glycosylation of apo(a) although the mechanisms underlying this observation remain to be determined in the context of angiogenesis.

## Introduction

Elevated plasma concentrations of lipoprotein(a) (Lp(a)) are recognized as an important emerging risk factor for the development of cardiovascular disease [1]. Lp(a) differs from low density lipoprotein (LDL) in that it contains a unique glycoprotein component apolipoprotein(a) (apo(a)), covalently linked to the apoB-100 moiety of LDL [2]. Apo(a) shares a high degree of homology with the serine protease zymogen plasminogen [3], and confers a number of unique functions to Lp(a) [4]. Apo(a) contains tandem repeats of a sequence that is very similar to the kringle (K) 4 domain of plasminogen, followed by sequences that are highly homologous to the plasminogen K5 and protease domains [3]. In apo(a), the plasminogen K4-like domains are further classified as apo(a) kringle IV types 1–10 (KIV_1_ to KIV_10_) based on amino acid sequence [3]. The KIV_2_ domain is present in a variable number of identically repeated copies, which gives rise to Lp(a) isoform size heterogeneity [5], [6]; there is a single copy of each of the other nine kringle IV domains in the apo(a) molecule [5]. KIV_10_ contains a strong lysine-binding site (LBS) that is required for the lysine-dependent binding of Lp(a)/apo(a) to physiological substrates such as fibrin [7], [8]. The LBS in this domain was most recently reported to be crucial for the apo(a)-mediated increase in endothelial cell contraction and permeability [9]. The KV domain of apo(a) has been shown to contribute, in part, to the ability of apo(a) to inhibit plasminogen activation system on the fibrin surface [10], and may also participate in maintaining the conformation of the apo(a) molecule [11]. Most recently, it has been suggested that the KV domain of apo(a) contains oxidized phospholipid covalently linked to specific lysine residues [12].

Physiological angiogenesis is a highly regulated, exquisitely coordinated balance of angiogenic and anti-angiogenic (i.e., angiostatic) factors [13], and involves endothelial cell migration, proliferation and differentiation to form tube-like structures corresponding to capillary sprouts [14]. Key to this process is the requisite remodeling of the extracellular matrix, through the activity of matrix metalloproteases which are activated by plasmin. The process becomes uncontrolled under conditions of pathological angiogenesis such as occurs in atherosclerosis during collateral vessel growth [15]. Both angiostatin (a proteolytic fragment containing kringles 1–4 of plasminogen [16], as well as fragments containing the plasminogen kringle 5 sequence are well characterized with respect to their anti-angiogenic activity [17]. As such, the effect of apo(a), which contains a domains that are highly homologous to plasmingen kringles 4 and 5, on the process of angiogenesis has been extensively studied. However, the role of apo(a)/Lp(a) in this context remains highly controversial, as reflected by a number of conflicting reports in the literature. For example, findings range from a positive effect using Lp(a) [18], to no effect using full-length recombinant apo(a) [19] or an apo(a) fragment [20], to an inhibitory effect using either full-length recombinant apo(a) [20], [21] or different fragments of apo(a) [21]–[24]. Fundamental differences exist between these studies, including sources of purified apo(a)/Lp(a) (from bacterial versus mammalian recombinant expression systems, for example) and choice of models used to identify and quantify angiogenesis. Importantly, the bacterially-expressed apo(a) lacks glycosylation modification which may influence the variable results obtained in some models of angiogenesis. Schulter and colleagues [21] reported that apo(a) was able to inhibit endothelial cell tube formation in fibrin gels; this group speculated that the effect of apo(a) on angiogenesis might result from its capability to inhibit plasminogen activation system, with potential consequences for extracellular matrix (ECM) remodeling. Although we and others have demonstrated that apo(a) can inhibit the plasminogen activation system in the context of fibrin [25], Schulter and colleagues reported that apo(a) decreases urokinase-type plasminogen activator (uPA) production in cultured human umbilical vein endothelial cells (HUVECs), thus reflecting the potential for an indirect effect of apo(a) on the plasminogen activation system [21].

The activation phase of angiogenesis involves several steps in which proteolytic enzymes such as plasmin play key roles [26]. These include extravascular fibrin deposition following an increase in vascular permeability, degradation of the basement membrane, and migration of the cells through the ECM, with concomitant remodeling of the matrix. Proteases are believed to play important roles in angiogenesis not only due to their ability to cleave the ECM, thereby allowing cells to migrate and invade, but also because of their potential to switch on neovascularization through activation, liberation and modification of pro-angiogenic factors [27]. Thus, the ability of Lp(a)/apo(a) to inhibit the plasminogen activation system [10], [25] provides a basis to hypothesize that Lp(a)/apo(a) may have an inhibitory effect on angiogenesis.

**Figure 1:**
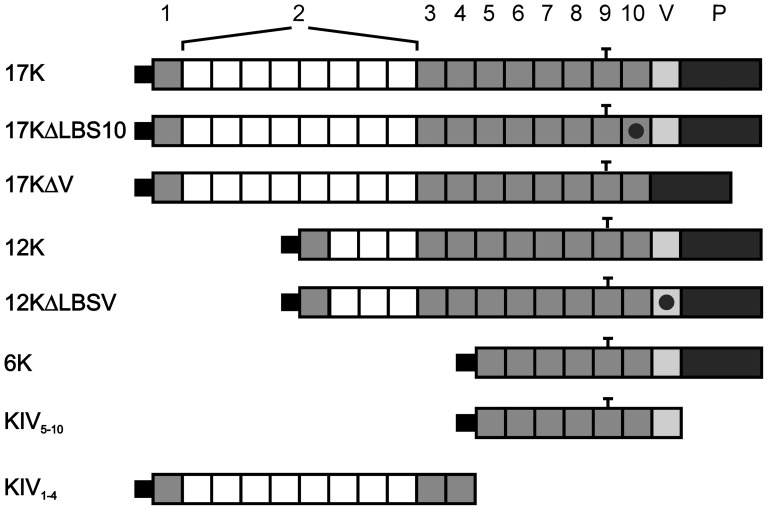
Recombinant apo(a) variants used in the study. The topology of the r-apo(a) variants used is shown in this schematic diagram. The top line represents the organization of the 17K r-apo(a) variant, which corresponds to a physiological apo(a) isoform and includes all 10 types of kringle IV sequences present in apo(a) isoforms, as well as the kringle V (V) and protease-like (P) domains. The black dot within a kringle denotes the presence of an amino acid substitution that inactivates the lysine binding site (LBS) in that kringle. The bar above KIV_9_ denotes the unpaired cysteine residue in this kringle that mediates covalent attachment to apoB-100 in LDL.

To address this hypothesis, we employed a fibrin gel matrix as has been previously reported [21], [28] to examine the effect of recombinant apo(a) (r-apo(a)) on the process of tube formation in human umbilical vein endothelial cells (HUVECs) mediated by vascular endothelial growth factor (VEGF) or angiopoetin-1 (Ang-1), and to explore a connection with the plasminogen activation system in this regard. Additionally, in order to provide clues about the mechanism of apo(a) action, we sought to identify the functional domain(s) of apo(a) that mediate(s) its effects on angiogenesis by comparing full length apo(a) with apo(a) fragments and mutated variants of apo(a) (Figure 1). Finally, in an attempt to resolve some of the contradictory reports in the literature concerning the nature of the role of Lp(a)/apo(a) in angiogenesis that have arisen due to the use of non-glycosylated apo(a) we examined the effect of deglycosylation of full-length r-apo(a) expressed in mammalian cells on this process. Our results indicate key roles for apo(a) kringle V and glycosylation modification in the ability of apo(a) to inhibit angiogenesis.

## Materials and Methods

### Materials

F-12K medium and fetal bovine serum (FBS) were obtained from the American Type Culture Collection (ATCC). Antibiotic solution (10,000 I U/mL penicillin, 10,000 µg/mL streptomycin, 25 µg/mL amphotericin B) was purchased from ICN Pharmaceuticals. EC growth factor (ECGF) was obtained from Roche. Vascular endothelial growth factor (VEGF) and angiopoietin-1 (Ang-1) were purchased from R&D. Heparin, thrombin, and *Clostridium perfringens* sialidase (EC 3.2.1.18; CPS) were purchased from Sigma. MMP-2, MMP-9, urokinase-type plasminogen activator (uPA), and human fibrinogen were from Calbiochem. Plasmin was obtained from Haematologic Technologies Inc.

### Expression and Purification of Recombinant Apo(a) Variants

The r-apo(a) variants used in the present study are shown schematically in Fig. 1. The construction and expression of these r-apo(a) variants has been described previously [9], [10], [29]–[32]. All r-apo(a) variants were purified from the conditioned medium (CM) of stably expressing human embryonic kidney (293) cell lines by lysine-Sepharose affinity chromatography as previously described [29], with the exception of apo(a) KIV_1–4_ [9]. Protein concentrations for each purified r-apo(a) variant were determined by absorbance measurements at 280 nm (corrected for Rayleigh scattering) using the molecular weights and extinction coefficients reported previously [10], [31], [32]. All proteins were assessed for purity by analysis on SDS-PAGE using a 4–20% gradient gel followed by silver staining. Purified proteins were stored in aliquots at −70°C prior to use.

### Cell Culture

Human umbilical vein endothelial cells (HUVECs) were obtained from ATCC (# CRL-1730). HUVECs were cultured in F-12K medium supplemented with 15% FBS, 1% antibiotic solution, heparin (100 µg/mL), and ECGF (20 µg/mL). Cells were maintained at 37°C in 5% CO_2_. Cells between passages 13 and 18 were used in these experiments as previously described [28]. For some experiments, fetal bovine serum was depleted of plasminogen by passage over a lysine-Sepharose column as previously described [33].

### Preparation of Fibrin Gels

Endotoxin- and plasminogen-free human fibrinogen was prepared as previously described [34]. Fibrinogen was dissolved in F-12K medium at a concentration of 5 µg/mL and thrombin was added to the solution to polymerize the gel. Fibrin gels were subsequently soaked in cultured medium for 1 h at 37°C to inactivate the excess thrombin. HUVECs were plated on the surface of the three-dimensional matrix and cultured in the absence or presence of reagents under study.

### In Vitro Angiogenesis Assays

HUVECs were cultured on the fibrin matrix in the presence of the treatments indicated in the text and in the respective figure legends. At the end of 24 h or 48 h incubation with the indicated treatment, images were captured using a Nikon digital camera (CoolPix 995) and these were analyzed by Image J (Wright Cell Imaging Facility). For each of six randomly pre-selected fields, the total length of capillary-like structures >30 µm was determined; the total area of the residual HUVEC monolayer was determined for the same field. The differentiation index (DI) was calculated as the ratio of total tube length to cell area for each field examined [28].

### Deglycosylation of 17K r-apo(a)

17K r-apo(a) (10 µg) was incubated with CPS (0.05 units) in 100 µL of phosphate buffer (pH 7.3) overnight at 37°C. The reaction was stopped by heating the reaction mixture at 80°C for 30 min. Deglycosylation of r-apo(a) was confirmed by SDS-PAGE followed by silver staining.

### Statistical Methods

Comparisons between data sets were performed using the Student's t-test (assuming equal variances). Statistical significance was presumed at *p*<0.01.

## Results

### Apo(a) Inhibits angiopoieten-1 and VEGF-mediated Tube Formation in HUVECs

**Figure 2:**
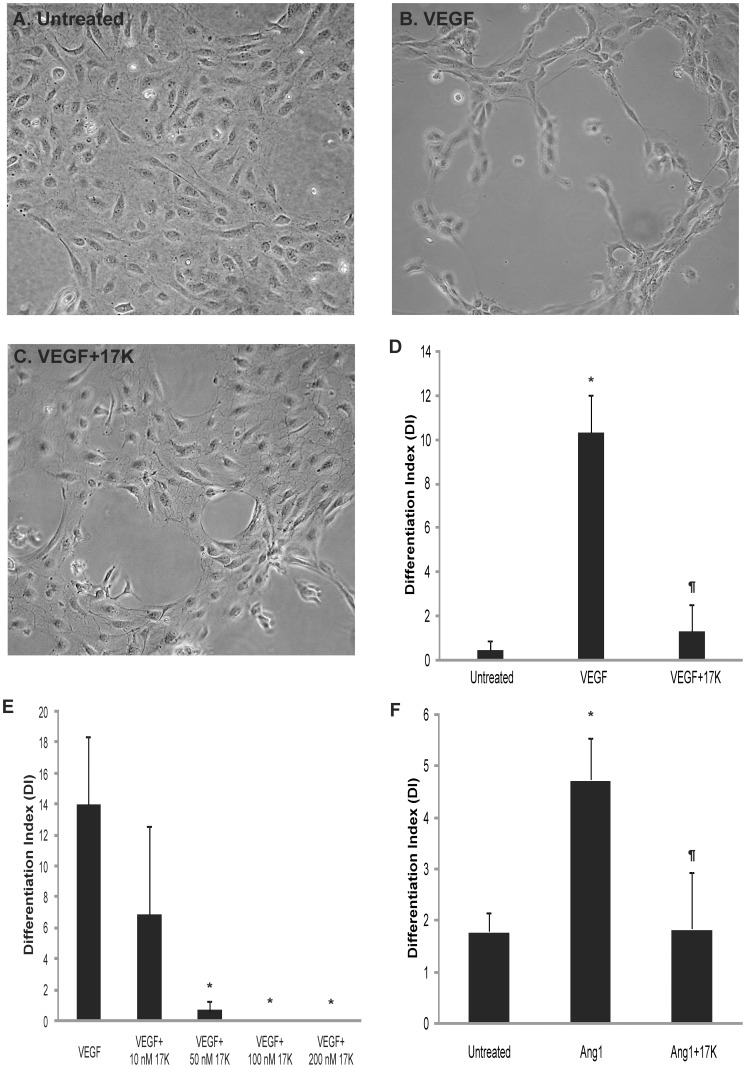
17K r-apo(a) blocks capillary-like tube formation induced by VEGF and angiopoietin-1 in three-dimensional fibrin matrices. The tube formation assay was conducted essentially as described by Babaei and colleagues [28]. HUVECs were cultured on fibrin matrices in the absence (**A**) or presence (**B**) of VEGF (20 ng/mL), or with VEGF and 100 nM 17K r-apo(a) (**C**). After 24 hours, light microscopy images of six randomly preselected fields for each treatment were taken using a digital camera (10×) and analyzed by a computer-assisted morphometric analysis system. For each image, the differentiation index (DI) was calculated as the ratio of total tube (capillary-like structures >30 mm) length divided by the total area of residual EC monolayer for the same field. **D–F**: Quantitative analysis of capillary-like network formation calculated as DI. Scale bars represent the mean ± standard deviation of the DI value obtained for each culture well from six independent images; data shown are representative of three independent experiments. Asterisks: p<0.01 versus untreated; pilcrow sign (¶): p<0.01 versus VEGF or Ang-1 (100 ng/mL), as appropriate.

We initiated our investigation by examining the ability of 17K r-apo(a), a physiologically-relevant form of apo(a), to modulate the *in vitro* tube formation process in cultured human umbilical vein endothelial cells. For this study, two well-established pro-angiogenic factors, vascular endothelial growth factor (VEGF) and angiopoietin-1 (Ang-1), were employed to stimulate angiogenesis in a 3-dimentional fibrin gel matrix as previously described [21], [28], [34]. It was demonstrated that while untreated HUVECs remained as a confluent monolayer, HUVECs treated with 20 ng/mL VEGF developed extensive capillary-like tube structures (Figure 2A,B). Addition of 17K r-apo(a) clearly inhibited the tube formation induced by VEGF (compare Figure 2C to Figure 2B). Quantitative analysis of the capillary network formation confirmed that VEGF significantly stimulated HUVEC tube formation, and that this stimulation was significantly suppressed by 17K r-apo(a) at concentrations greater than 50 nM (Figure 2DE). A similar pattern of results was obtained when 100 ng/mL Ang-1 was used to stimulate HUVEC tube formation (Figure 2F).

### Plasmin, but not MMPs, Rescues VEGF-Induced Tube Formation in the Presence of Apo(a)

**Figure 3:**
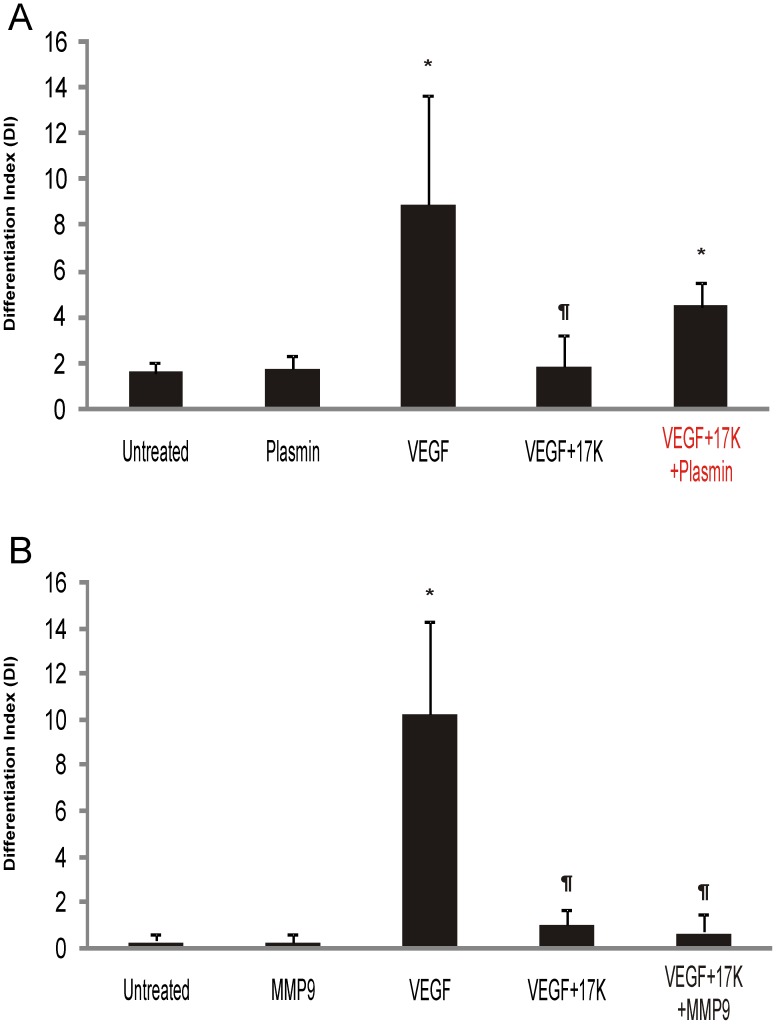
The effects of plasmin and MMP-9 on the 17K apo(a)-mediated inhibition of HUVEC tube formation. HUVECs were cultured on fibrin matrices with the indicated treatments (VEGF: 20 ng/mL; 17K r-apo(a): 100 nM; plasmin (**A**): 50 nM; MMP-9 (**B**): 8 nM). Tube formation was measured as described in the legend to Figure 2. Scale bars represent the mean ± standard deviation of the DI value obtained for each culture well from six independent images; data shown are representative of three independent experiments. Asterisks: p<0.01 versus untreated; daggers: p<0.01 versus VEGF.

Considering the key role of the plasminogen activation system in angiogenesis and the reported ability of apo(a) to interfere with this system in the context of both fibrin and cell surfaces [10], [25], [33], the role of the plasminogen activation system in apo(a) inhibition of tube formation was examined. We chose plasmin and matrix metalloproteinases (MMP-2 and MMP-9) for assessment of their ability to mitigate the inhibitory effects of apo(a) as these proteases have been implicated in regulating angiogenesis [35], [36]. While neither plasmin nor MMP-9 at the concentrations used in our study caused significant changes in the basal level of HUVEC tube formation (Figure 3A and 3B, respectively), the inhibitory effect of 17K r-apo(a) on VEGF-induced tube formation was significantly rescued by the addition of plasmin (Figure 3A) but not by the addition of MMP-9 (Figure 3B) or MMP-2 (data not shown).

**Figure 4:**
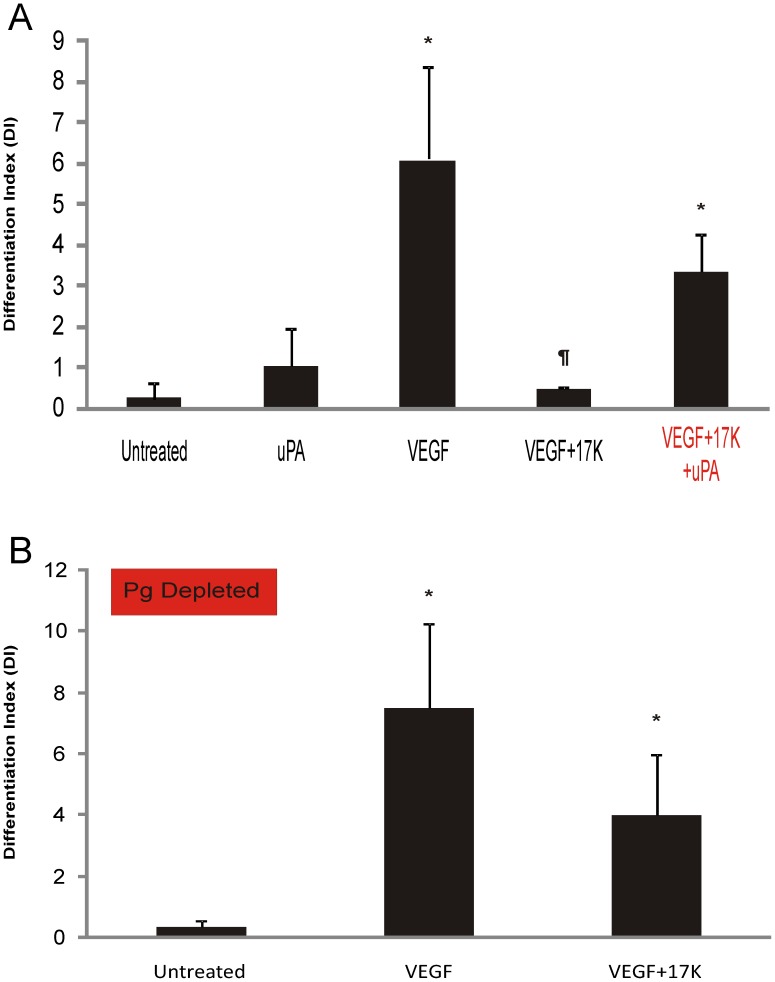
The effects of addition of uPA and depletion of plasminogen on the 17K apo(a)-mediated inhibition of HUVEC tube formation. HUVECs were cultured on fibrin matrices with the indicated treatments (VEGF: 20 ng/mL; 17K r-apo(a): 100 nM; uPA (**A**): 0.5 nM). Experiments in **B** were conducted using plasminogen-depleted serum. Tube formation was measured as described in the legend to Figure 2. Scale bars represent the mean ± standard deviation of the DI value obtained for each culture well from six independent images; data shown are representative of three independent experiments. Asterisks: p<0.01 versus untreated; daggers: p<0.01 versus VEGF.

To further examine the role of the plasminogen activation system in mediating the anti-angiogenic effects of apo(a), additional components of this system were tested. First, we showed that uPA was, like plasmin, capable of rescuing the anti-angiogenic effects of 17K r-apo(a) (Figure 4A). Next, we employed serum that had been depleted of plasminogen in the cell growth medium for the tube formation assays. Under these conditions, although 17K r-apo(a) did decrease tube formation, the difference between VEGF-induced angiogenesis in the absence or presence of 17K apo(a) was no longer significant (Figure 4B). It is noteworthy, however, that the absence of plasminogen generally suppresses the angiogenic potential of HUVECs in this system. In order to achieve the same extent of tube formation as was observed in the presence of plasminogen (Figure 4A), it was necessary to incubate the cells for 48 h instead of 24 h, and to use a five-fold higher concentration of VEGF (Figure 4B). Nonetheless, these data are not inconsistent with the findings of Schulter and colleagues showing that apo(a) decreases uPA secretion from endothelial cells [21].

### The Apo(a) KV Domain is Required for Apo(a) Inhibition of VEGF-Induced Tube Formation

**Figure 5:**
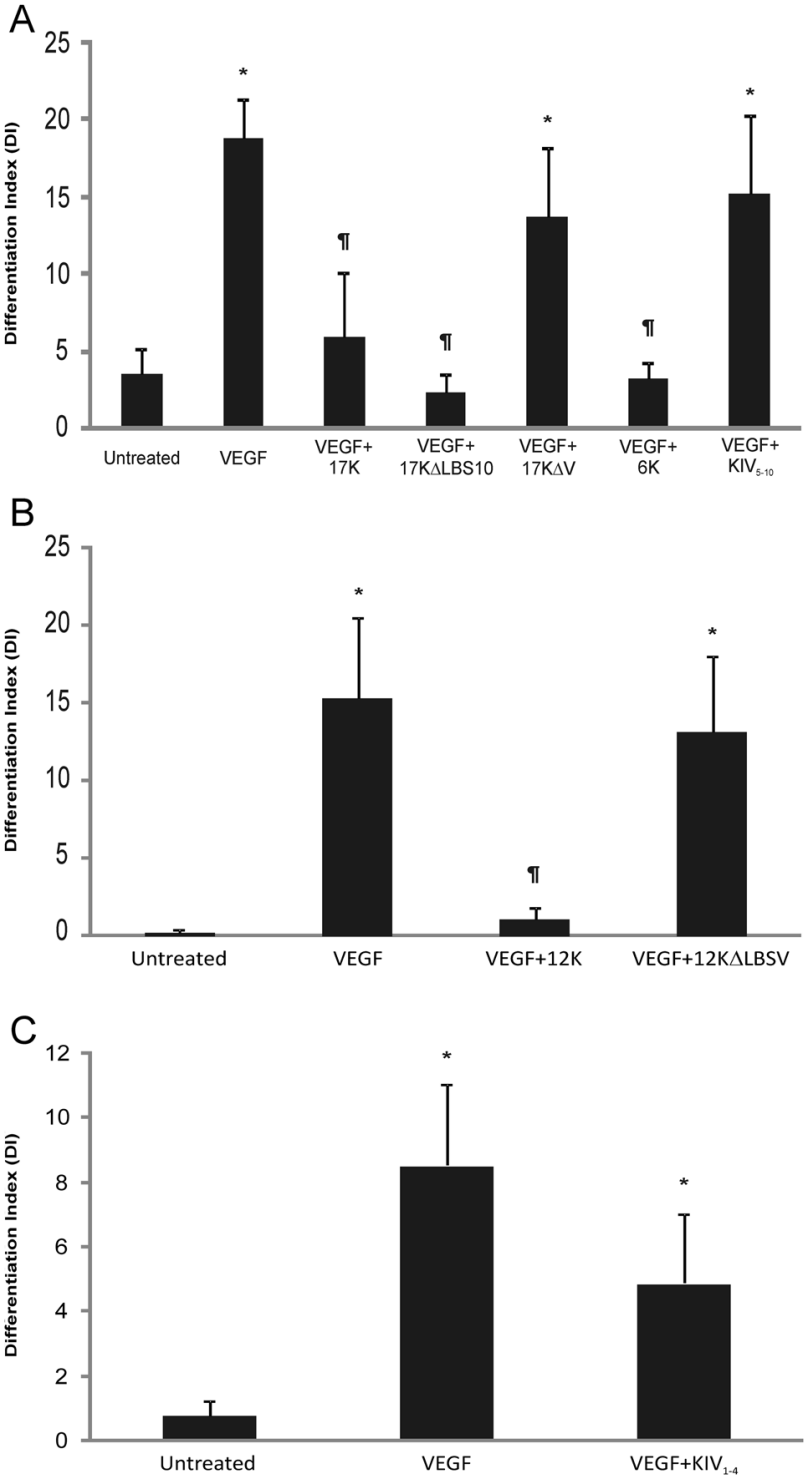
Effects of different r-apo(a) variants on HUVEC tube formation. HUVECs were cultured on fibrin matrices with the indicated treatments (VEGF: 20 ng/mL; 17K r-apo(a) and all other variants: 100 nM). Tube formation was measured as described in the legend to Figure 2. Scale bars represent the mean ± standard deviation of the DI value obtained for each culture well from six independent images; data shown are representative of three independent experiments. Asterisks: p<0.01 versus untreated; daggers: p<0.01 versus VEGF.

Identification of the functional domain that is responsible for the inhibitory effect of apo(a) on HUVEC tube formation was pursued through analysis of tube formation after treatment with a series of r-apo(a) variants (Figure 1). First, we examined the ability of 17KΔLBS_10_ and 17KΔV variants to inhibit VEGF-induced tube formation using the methodology described above. While 17KΔLBS_10_ contains an Asp to Ala substitution at amino acid position 57 in the KIV_10_ domain, which disrupts the strong LBS present in this kringle, the 17KΔV variant completely lacks sequences corresponding to the KV domain. The data in Figure 5A clearly show that the KV domain, but not the strong LBS in KIV_10_, is required for the apo(a)-mediated inhibition of VEGF-induced tube formation in HUVECs. The inhibitory effect of the KV domain in apo(a) in this process was further confirmed in the tube formation assay by directly comparing the 6K apo(a) variant (containing KIV_5–10_ as well as the KV and protease-like domains) with the KIV_5–10_ apo(a) variant (Figure 5A). In order to determine a possible role for the weak LBS in KV, a 12K apo(a) variant was directly compared with 12K containing a mutation in the weak LBS of kringle V (Asp to Ala substitution at position 57 of this kringle (12KΔLBSV) [29], [37] (Figure 5B). While both 6K and 12K variants retain the inhibitory effect on tube formation, the two corresponding variants that contain either no KV sequence, or a KV domain with a mutation in the lysine-binding site (KIV_5–10_ and 12KΔLBSV, respectively) are unable to inhibit VEGF-stimulated HUVEC tube formation. Finally, we tested a recombinant apo(a) variant corresponding to the N-terminal half of apo(a) (KIV_1–4_) and demonstrated that it had no significant effect on VEGF-induced HUVEC tube formation (Figure 5C).

### The Glycosylation Modification of Apo(a) is Required for its Ability to Inhibit Tube Formation

**Figure 6:**
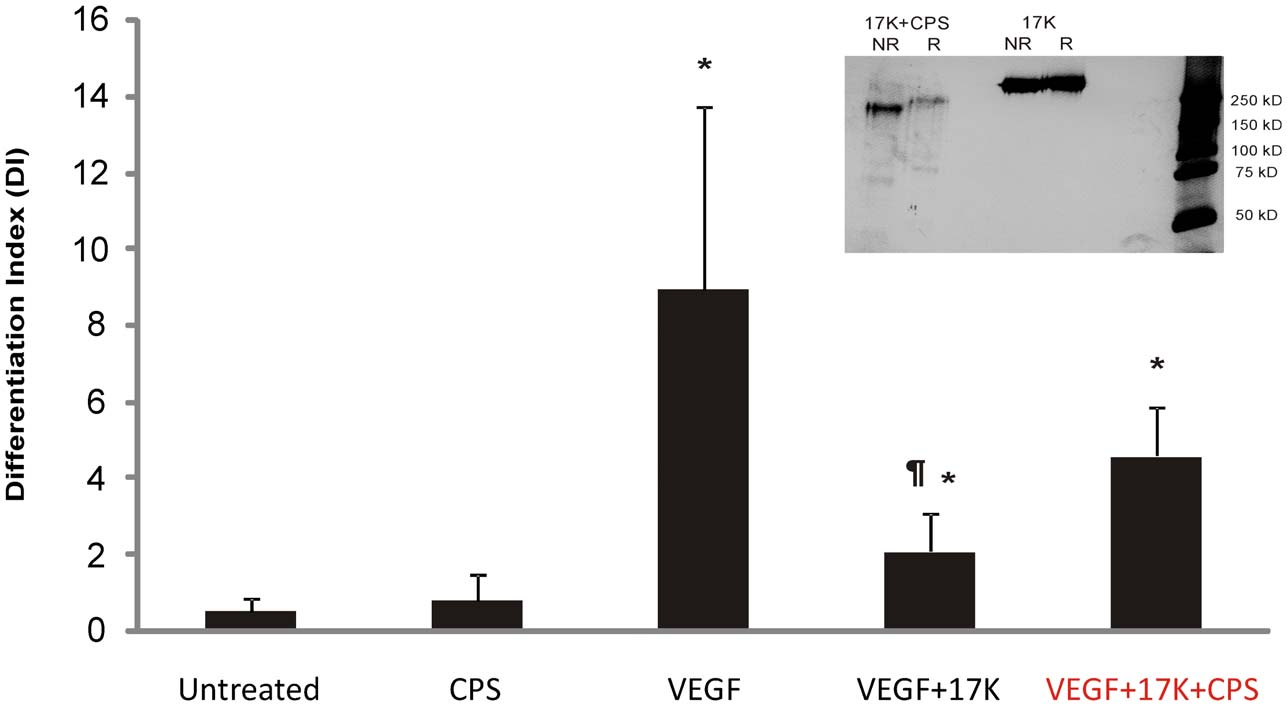
Effect of deglycosylation on the ability of r-apo(a) to inhibit HUVEC tube formation. 17K r-apo(a) was incubated with CPS overnight at 37°C. The deglycosylated material was subjected to SDS-PAGE under non-reducing (NR) or reducing conditions, followed by silver-staining (inset to graph). HUVECs were cultured on fibrin matrices with the indicated treatments (VEGF: 20 ng/mL; 17K r-apo(a), either untreated or treated with CPS: 100 nM; CPS: 0.14 U/mL, equivalent to the amount of CPS added along with the CPS-treated apo(a)). Tube formation was measured as described in the legend to Figure 2. Scale bars represent the mean ± standard deviation of the DI value obtained for each culture well from six independent images; data shown are representative of three independent experiments. Asterisks: p<0.01 versus untreated; daggers: p<0.01 versus VEGF.

Human apo(a) is a highly glycosylated protein (28% carbohydrate by weight) [30], [38]. Apo(a) is rich in sialic acid [30], [38] that is present on multiple *N*- and *O*-linked glycosylation sites present in the molecule [3], [39]. One possible explanation for the disagreement regarding the role of apo(a)/Lp(a) in angiogenesis is that different studies have used apo(a)/Lp(a) obtained from a variety of sources, including transfected mammalian cell lines, human plasma, and plasma from transgenic mice, as well as bacterially-expressed apo(a) fragments [18]–[24]. It is likely that these different sources of apo(a)/Lp(a) are variable in their glycosylation modification, which may affect the observed function of apo(a)/Lp(a) in the different angiogenesis models used. To address the importance of the glycosylation of apo(a) in mediating its anti-angiogenic effect, 17K apo(a) was pre-treated with CPS glycosidase to remove terminal sialic acid. The CPS-treated 17K exhibited increased electrophoretic mobility on SDS-PAGE, and migrated to an extent comparable with its theoretical molecular weight of 278,000 [30] thereby suggesting complete reaction with CPS (Figure 6, inset). When the desialylated 17K-r-apo(a) was tested in tube formation assays, it was found to be unable to significantly inhibit VEGF-induced tube formation (Figure 6). The 17K r-apo(a) used for comparison in this experiment was mock-treated with CPS under the same reaction conditions (i.e., 12 h at 37°C) to confirm that the lack of activity of desialylated recombinant apo(a) could not be attributed to the effects of extended incubation of the protein at 37°C.

## Discussion

Identification of a physiological role for Lp(a) has remained elusive, largely because of the absence of an animal model expressing this lipoprotein that is amenable to genetic manipulation [40]. The homology of apo(a) to plasminogen [3] and, hence, to angiostatin has inspired many investigators to study a role for Lp(a) in angiogenesis; this has been further encouraged by the identification of a strong anti-angiogenic role for plasminogen kringle 5. A variety of *in vitro* and *in vivo* studies have led to conflicting results, with both pro- and anti-angiogenic effects reported for Lp(a)/apo(a), as well as several reports of no effect whatsoever [18]–[24]. Although several groups have provided strong evidence for a pro-angiogenic role for Lp(a)/apo(a), a study in transgenic mice expressing human Lp(a) (with human apo(a) and apoB expressed from separate transgenes) reported that Lp(a) inhibited collateral formation in a model of peripheral arterial disease [41]. Studies by several groups have focused on the role of specific kringles in inhibiting angiogenesis and tumour growth [20], [22], [42]. A consistent feature of these studies is that all apo(a) variants that inhibit angiogenesis or elicit pro-angiogenic responses in the endothelium contain the KV domain of the molecule. However, the molecular basis for these observations is unclear, particularly in light of the non-physiological nature of the small kringle fragments used in these studies.

The present study was motivated to advance the previous work by Schulter and colleagues [21] using a similar fibrin matrix tube formation assay in endothelial cells as they reported. We have corroborated their findings using different angiogenic factors (VEGF and Ang-1 in our study versus basic fibroblast growth factor and tumor necrosis factor-α as they reported). Additionally, our studies provide new knowledge by identifying a critical role of the apo(a) KV domain and its weak lysine-binding site in inhibiting tube formation in this system. Interestingly, we observed no effect of KIV_1–4_ on inhibition of angiogenesis in our studies. This is not consistent with the findings of Schulter and colleagues [21] which suggest that the urinary fragments of apo(a), derived from the amino-terminal half of the molecule [43] can inhibit angiogenesis in their system. The basis of this inconsistency is not clear, but may relate to modification of these fragments as they are proteolytically processed and excreted in the kidney or the presence of some contaminating factor in the fragments isolated from urine.

The plasminogen activation system comprises plasminogen (the proenzyme), plasmin (the active enzyme), several activators and inhibitors that regulate the formation of plasmin and its activity, and a surface (either fibrin or cells) that serves to localize and accelerate plasminogen activation. The serine protease plasmin has been long established as an important player in promoting angiogenesis by pericellular degradation of components of the surrounding ECM, including fibronectin, laminin, and proteoglycans as well as through activation of key matrix metalloproteases [44]. In the context of our experimental approach, endothelial cell tube formation has been reported to be dependent on plasmin activity [45], [46]. Moreover, fibrin degradation products generated from fibrin cleaved by plasmin can also induce angiogenesis [47], [48]. In the current study, the dependence of apo(a) inhibition of tube formation in HUVECs on its ability to interfere with the plasminogen activation system was demonstrated by ‘activating’ (adding plasmin or uPA) or ‘deactivating’ (removing plasminogen from the serum in the medium) the plasminogen system. Under both conditions, 17K r-apo(a) was impaired in its ability to inhibit the development of the VEGF-induced tubular network in HUVECs. Notably, both plasmin and uPA alone at the concentrations tested did not increase the extent of HUVEC tube formation. As such, although plasmin is considered to be indispensable for tube formation on a fibrin matrix, it is not sufficient to induce angiogenesis on its own. On the other hand, the pro-angiogenic property of uPA is well-established [49]. Indeed, when added at higher concentrations (2 and 5 nM), uPA itself was able to induce HUVEC tube formation (data not shown).

In addition to the components of the plasminogen activation system, MMPs represent another important family of proteases that is heavily involved in angiogenesis regulation, especially MMP-2, -9, and -14 [50]. We have tested both MMP-2 and MMP-9 in our study and, unlike plasmin, neither of them were able to restore tube formation to levels observed prior to 17K apo(a) treatment. Thus, although apo(a) may decrease plasmin formation, the effect of apo(a) on inhibiting angiogenesis is not through a decrease in MMP activation.

It is noteworthy that we have identified the KV domain as being responsible for apo(a) inhibition of tube formation, considering that this domain shares approximately 80% amino acid sequence identity with the KV domain in plasminogen [3]. The anti-angiogenic property of the plasminogen KV domain was discovered shortly after angiostatin was identified as a physiologically-relevant inhibitor of angiogenesis [51], [52]. Moreover, the apo(a) KV domain has been expressed recombinantly, and has been shown to inhibit both *in vitro* and *in vivo* angiogenesis [23]. With respect to plasminogen activation system inhibition, our laboratory has previously identified a contributing role for the apo(a) KV domain in this process utilizing a similar pool of r-apo(a) variants to that used in the present study [10]. Interestingly, although our previous study also identified the KIV_10_ domain and the amino-terminal half of apo(a) as necessary elements for apo(a) to maximally inhibit *in vitro* fibrin-dependent plasminogen activation system [10], apo(a) variants lacking these domains (17KΔLBS_10_ and 6K, respectively) were both able to inhibit HUVEC tube formation in the current study. Either the domains in apo(a) that influence plasminogen activation in the current milieu containing HUVECs may be distinct, or the effect of apo(a) is entirely attributable to a decrease in uPA secretion [21]. The domains in apo(a) that mediate this latter effect remain unknown.

Apo(a) also affects vascular smooth muscle cell responses through an effect on the plasminogen activation system. We and others have shown that apo(a) stimulates smooth muscle cell proliferation and migration by interfering with plasmin-mediated conversion of latent TGF-β to its active form [33], [53]. We proposed that this resulted from inhibition of pericellular plasminogen activation, and described an essential role for apo(a) KIV_9_ in this process. Since the domains in apo(a) that modulate HUVEC tube formation in the present study are different, it is likely that the effects of apo(a) in this context also reflect inhibition of fibrin-stimulated plasminogen activation, versus cell-surface-stimulated plasminogen activation.

In an earlier study from our laboratory [29], we reported that apo(a) is able to stimulate HUVEC migration and proliferation. While this result may be interpreted as in conflict with our current finding that apo(a) inhibits tube formation, there are some important differences that need to be emphasized. First, the migration and proliferation experiments were not done in the presence of VEGF or Ang-1, and were carried out on cells grown on plastic versus in fibrin matrices [29]. Second, the domains in apo(a) that mediate these effects appear to be different. In the current study, removal of KV or its LBS abolished the effect of apo(a) on tube formation, while mutation of the strong LBS in KIV_10_ had no effect (Figure 5). In our earlier study, a partial or complete abolishment of the effects of apo(a) were achieved by removal of KV or the KIV_10_ LBS [29]. Thus, the domain requirements for the two effects are different. Interestingly, we had found earlier that apo(a) stimulates phosphorylation and thus activation of the MAP kinases p38 and ERK1/2 [29]. These two kinases have been shown to have distinct roles in regulating HUVEC tube formation in collagen matrices [54]. Thus, we would conclude that the effects of apo(a) on HUVEC migration/proliferation and tube formation represent distinct phenomena possibly involving underlying differences in signal transduction pathway activation. Further work will be required to resolve the ramifications of these various effects *in vivo*.

A recent study has shown that a peptide derived from apo(a) KV can, at high concentrations, inhibit angiogenesis and tumor growth [55]. Interestingly, this peptide does not span the mutation abolishing the weak LBS in KV that, in our study, is required for inhibiting tube formation. Therefore, it is possible that this peptide inhibits angiogenesis through a mechanism distinct from that utilized by the intact apo(a) molecule. Alternatively the isolated KV domain, shown by others to inhibit angiogenesis [23], itself uses a different cellular mechanism. Only further mechanistic studies will be able to distinguish between these possibilities. It is noteworthy that isolated KV and fragments encompassing KIV_9_-KV or KIV_10_-KV also inhibit endothelial cell migration and proliferation [22], [56]. Our previous studies have shown that full-length apo(a) can, in fact, stimulate both of these processes in HUVECs [29]. Moreover, we found that removal of the KV domain or mutation of the LBS in KIV_10_ or KV was sufficient to abolish the effect of apo(a) on proliferation and to greatly diminish the effect of apo(a) on migration [29]. We did not test small fragments of apo(a) in these studies. Finally, the KV peptide interferes with the Src/ERK pathway whereas our studies using intact apo(a) showed that apo(a) stimulates Src and ERK phosphorylation [29]. Therefore, it appears that the effects of isolated apo(a) fragments on endothelial cells are distinct from those of full-length apo(a). It should be noted that the 17K r-apo(a) that we employed in our studies represents a physiologically-relevant apo(a) isoform. Moreover, our apo(a) variants are glycosylated, unlike the KV peptide or bacterially-expressed KIV_9_-KV fragments. Notably, we found that deglycosylation of apo(a) abolished its ability to inhibit tube formation, suggesting that the glycans on apo(a) may play a direct or indirect (such as influencing apo(a) conformation) role on the plasminogen activation system in this context.

**Figure 7:**
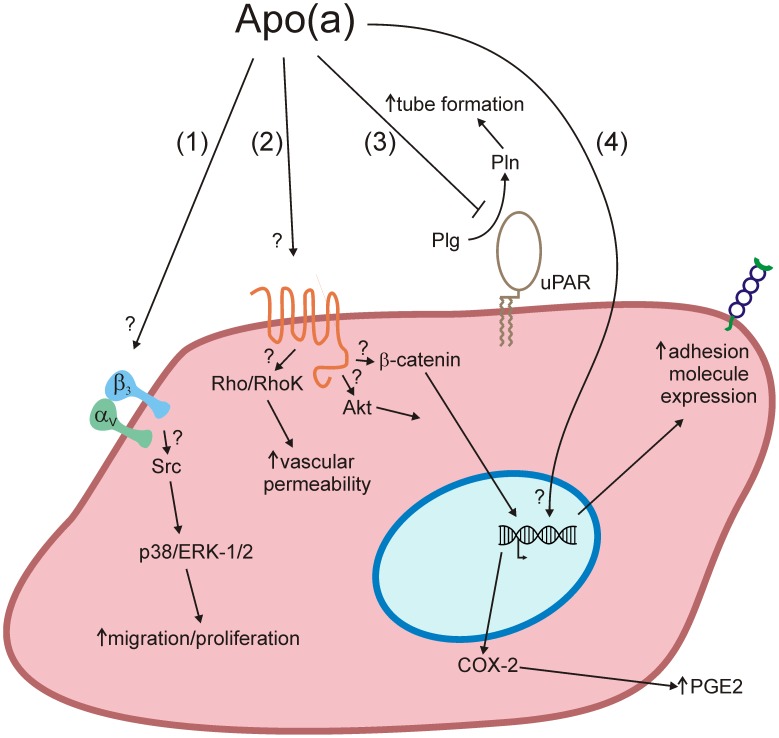
Complex effects of apo(a) on endothelial cell function. (1) Through a mechanism involving, in a manner yet to be defined, integrin α_V_β_3_, apo(a) induces Src and MAP kinase activation that results in increased migration and proliferation [29]. (2) Through binding to a cell surface receptor that is currently undefined, apo(a) results in activation of Rho, which through its effector Rho kinase (RhoK) ultimately results in actin/myosin stress fiber formation and increased endothelial cell contraction and vascular permeability [9]. In addition, through the dissociation of β-catenin form adherens junction and the promotion of β-catenin nuclear translocation through activation of Akt, apo(a) elicits an increase in the expression of cyclooxygenase-2 which results in increased synthesis of prostaglandin E2 (PGE_2_) (unpublished observations). (3) Apo(a) inhibits pericellular plasminogen activation that is likely dependent on uPA and the uPA receptor (uPAR), an effect that results in a decrease in tube formation (current study). (4) Through unknown mechanisms, apo(a) results in increases in the expression of several adhesion molecule genes including ICAM-1 and E-selectin [4].

Despite a profusion of potential mechanisms by which Lp(a) can harm the vasculature, the true biological role of Lp(a), if any, has remained elusive. Lp(a) is unusual in that its plasma levels vary over 1000-fold in the human population. It is important to bear in mind that in the current study apo(a) was primarily tested at a concentration of 100 nM (equal to a Lp(a) concentration of ∼8 mg/dL), which is well below the clinical risk threshold identified for Lp(a) (25–30 mg/dL [1]). At higher concentrations, it has been demonstrated by our group and others that apo(a) can induce alterations of endothelial function including expression of adhesion molecules [4],stimulation of rearrangements of the actin cytoskeleton leading to increased endothelial permeability and pro-inflammatory gene expression [9], and stimulation of migration and proliferation [29]. The complex effects of apo(a) on endothelial cells and the underlying mechanisms are summarized in Figure 7. The ability of apo(a) to inhibit tube formation within a fibrin-rich context at the concentrations tested in the present study might indicate an important physiological function of Lp(a) that predominates at sub-hazardous concentrations: inhibiting of angiogenesis. This may potentially protect the vasculature by maintaining the stability of atherosclerotic lesions. Indeed, angiogenesis can nurture plaque growth and also stimulate inflammation, which can promote the transition from stable to unstable atherosclerotic lesions [57].
